# An Apparently Practical Method for the Isolation of Pfeiffer’s Bacillus from Sputum

**Published:** 1919

**Authors:** 


***An Apparently Practical Method for the Isolation of Pfeiffer's Bacillus from Sputum.*** By E. E. Ecker. *The Journal of American Medical Association*, Nov. 2, 1918.

The difficulty encountered in isolating the B. influenzae from sputum is due to the fact that other organisms, such as pneumococci, staphylococci and the Micrococcus catarrhalis tend to obscure the colonies of influenza bacilli and overgrow them. In this connection, the author thought of using bile and bile salts which do not destroy the virulence of influenza bacilli, but have a dissolving action on pneumococci and other organisms. Bronchial secretions were mixed with a solution of sodium taurocholate, and the mixture was allowed to stand for at least 20 minutes. After exposure, streaks were made on human blood agar plates. After 20 hours incubation at 37 C., minute, colorless, transparent, droplet-like colonies were detected and differentiated. The organisms stain well with aqueous fuchsin, and measure about 0.5 to 0.6 micron by 0.2 to 0.3 micron. They are Gram-negative and form irregular clusters with no definite arrangements. They often give the appearance of B. coli. Subcutaneous injections of washings from slants into mice proved fatal to the animals within 6 or 7 hours. The bacilli were readily obtained from the heart blood of the animals.

